# New Insights on the Burst Release Kinetics of Spray-Dried PLGA Microspheres

**DOI:** 10.1021/acs.molpharmaceut.4c00686

**Published:** 2024-10-25

**Authors:** Kyprianos Michaelides, Mohamad Anas Al Tahan, Yundong Zhou, Gustavo F. Trindade, David J. H. Cant, Yiwen Pei, Pawan Dulal, Ali Al-Khattawi

**Affiliations:** † School of Pharmacy, Aston University, Birmingham B4 7ET, U.K.; ‡ Chemical and Biological Sciences Department, National Physical Laboratory, Hampton Road, Teddington TW11 0LW, U.K.; § aVaxziPen Limited, Milton Park, Abingdon, Oxfordshire OX14 4SA, U.K.

**Keywords:** PLGA microspheres, spray drying, burst release, chemical depth profiling, surface analysis

## Abstract

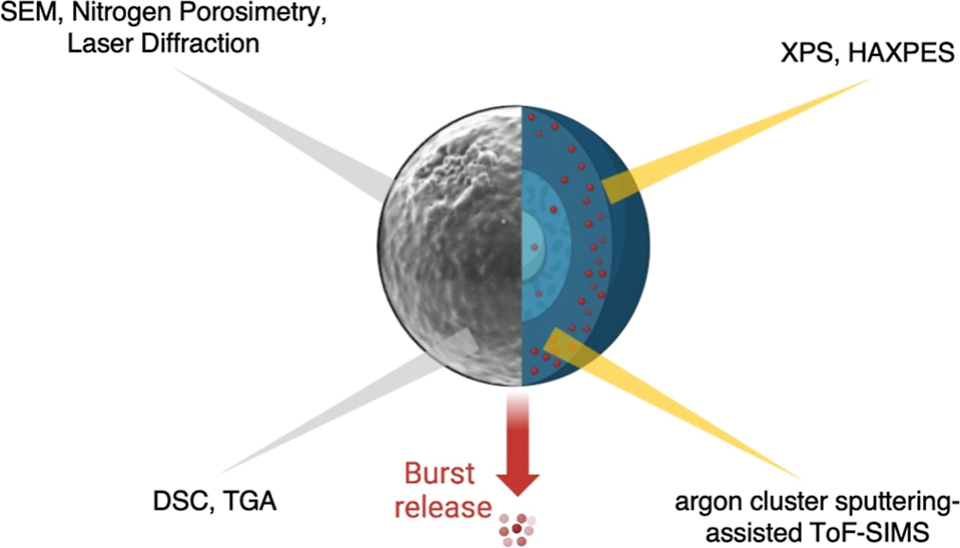

Spray drying is one of the leading manufacturing methods for active pharmaceutical ingredients (APIs) owing to its rapid, single-step, and cost-effective nature. It also has the capacity to generate microspheres capable of controlled release of APIs including biomolecules and vaccines. However, one of the key challenges of spray-dried formulations especially with poly(lactic-*co*-glycolic acid) (PLGA)-based controlled-release injectables is burst release, where a significant fraction of the API is released prematurely within a short period of time following administration, leading to detrimental impact on the performance and quality of end products. This study uses a model API, bovine serum albumin (BSA) protein, to identify the sources of burst release that may affect the kinetics and performance of long-acting injectable microsphere formulations. Spray-dried microspheres with various formulations (i.e., variable BSA/PLGA ratios) were characterized in terms of their morphology, particle size, surface area, thermal properties, moisture content, as well as chemical compositions and their distributions to investigate the impact of spray drying on the burst release phenomenon. The results suggest that a relatively high initial release (85%) observed is mainly attributed to the protein distribution close to the particle surface. Morphology analysis provided evidence that the microspheres retained their spherical structure during the burst release phase. X-ray photoelectron spectroscopy, hard X-ray photoelectron spectroscopy, and argon cluster sputtering-assisted time-of-flight secondary ion mass spectrometry analysis suggest an enrichment of PLGA on particle surfaces with buried BSA protein. The statistically significant difference in particle size and surface area between three different formulations may be responsible for an initial variation in release but did not seem to alter the overall burst release profile. Considering the suggested source of burst release, the two-fluid spray-drying method, characterized by a single liquid feed delivering a preprepared emulsion, generated matrix-type microspheres with a surface layer of PLGA, as evidenced by surface analysis. The PLGA surface layer proved to be prone to degradation and pore formation, allowing for faster diffusion of BSA out of the microspheres, resulting in a burst release. Increasing the polymer concentration did not seem to halt this process.

## 1 Introduction

Spray drying has been used in the pharmaceutical industry over the past decades for rapid drying, microencapsulation, protein/vaccine stabilization, and particle engineering.^1,2^ The microencapsulation and particle engineering capabilities of spray drying have been increasingly utilized for the preparation of controlled release formulations.^3^ Such formulations are particularly useful in delivering vaccines or other biomolecules to the body in the form of long-acting injectables.^4^ Various polymers, such as polycaprolactone (PCL) and chitosan, have been employed in conjunction with spray drying to produce microspheres and regulate the release of APIs.^5−7^ However, the utilization of PCL and chitosan in long-acting injectables has been restricted to a few studies, with poly(lactic-*co*-glycolic acid) (PLGA) emerging as the preferred choice. PLGA stands out as the predominant polymer employed in long-acting injections due to its well-established safety profile, biocompatibility, and biodegradability.^8,9^ In fact, spray-dried controlled release formulations containing PLGA microspheres for the peptides triptorelin pamoate and lanreotide acetate were approved by the United States Food and Drug Administration.^10^ However, one of the most common phenomena anticipated in controlled release formulations prepared by spray drying and other particle engineering methods is burst release.^11^ Specifically, a significant fraction of the encapsulated active pharmaceutical ingredients (API) is released within a short stretch of time (generally within hours or up to 1 or 2 days) following administration.^12^ In most cases, initial burst release is undesirable due to related toxicity issues or decrease in the overall duration of an API’s therapeutic effect. Occasionally, accurate control of this initial burst is necessary if an immediate pulse is required at the beginning of the regimen, e.g., a prime dose of a vaccine.^9^

The uncontrolled burst could be linked to the kinetics of droplet and particle generation during spray drying. Wet-bulb kinetics govern the rate of evaporation until the solute (e.g., PLGA) dissolved in the evaporating solvent approaches saturation.^3^ The formation of a crust resulting from solidification signals the start of the drying kinetics’ falling rate period.^13^ The crust temperature starts increasing and the rate of mass loss due to evaporation starts decreasing and becomes rate-limited by the ability of the solvent to diffuse through the crust.^14^ As the solvent evaporates during the process, a concentration gradient forms within the microspheres, leading to higher concentrations of the hydrophilic API near the surface. This migration of the API toward the surface is primarily driven by thermodynamic forces aiming to minimize interfacial energy. Additionally, the diffusion of solvents out of the microspheres can further contribute to this migration process.^15,16^ Accurate control of this process is of vital importance when spray-drying heat-sensitive biomacromolecules to prepare controlled release formulations with specific release requirements.

Various analytical expressions have been employed to describe burst release or overall drug release, as reviewed by other research groups in the past.^17,18^ The mathematical model developed by Corrigan and Li (2009) considers release in two separate phases: (1) The diffusion-controlled burst release of the drug which is close to or in contact with the release medium and (2) and the release of entrapped drug associated with bulk degradation of the polymer.^19^ For the initial phase, the following equation was proposed to explicitly express the burst release phenomena
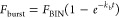
1where *F*_BIN_ is the fraction of API released by the burst release mechanism at infinite time and *k*_b_ is the first-order rate constant corresponding to burst release kinetics. The rate constant is equal to *DA*/*LV*, where *D* is the diffusivity of the API in an aqueous medium, *A* is the surface area of the polymer microparticle, *L* is the interfacial aqueous boundary layer thickness, and *V* is the volume of the polymer microparticle.^20,21^ Thus, a smaller surface area and larger volume of the microparticle would give a reduced *k*_b_ resulting in the suppression of burst release.

Some reviews have discussed the mechanisms responsible for, and proposed approaches to minimize, burst release from PLGA microspheres.^20,22,23^ Their main findings relate to the proportion of APIs on the surface of the microsphere, which can diffuse rapidly. Another contributor to the phenomenon is the microsphere size that seems to be inversely proportional to the burst effect.^20^ Smaller microspheres exhibit shorter diffusion distances and a higher surface area (*A*)-to-volume (*V*) ratio facilitating water absorption and, hence, increasing the burst release fraction (*F*_BIN_). Similarly, porous microspheres show higher burst effect compared to their nonporous counterparts because of their higher surface area and shorter diffusion distances.^24−26^ Furthermore, PLGA characteristics such as low lactic/glycolic ratio, low molecular weight, and noncapped ending groups lead to a less hydrophobic PLGA with more water absorption, promoting burst release profiles.^27^ The choice of organic solvent employed to dissolve the PLGA for spray drying has been shown to influence burst release;^28^ even though microspheres produced using dichloromethane (DCM) or acetone had similar particle sizes and protein surface enrichment, DCM-produced microparticles showed less burst release. This can be attributed to DCM being a better solvent for PLGA, leading to denser PLGA matrices capable of resisting collapse during early release phases.^29^ Various advanced analytical methods have also been utilized, including chemical and spatial analysis of protein-loaded PLGA microspheres. However, these methods were not directly associated with the microspheres’ release profile.^30^

The purpose of this study is to contribute to the understanding of the root causes of burst release from PLGA microspheres, which is a critical challenge in the development of long-acting injectables. Uniquely, the study focuses on linking the burst release behavior of these microspheres to an array of chemical and morphological attributes, thus providing a comprehensive structural understanding of the problem. PLGA microsphere formulations prepared by spray drying at different bovine serum albumin (BSA)/PLGA ratios were characterized using different techniques. Particle size distribution and surface area were characterized due to the potential important role in the initial burst effect. Furthermore, morphology evolution at different stages of protein release was investigated to reveal the impact of pore formation and erosion on the burst phase and overall release profiles. Moreover, surface chemical analysis and chemical depth profiling were used to understand the distribution of key components within particle samples, which can play a significant role in the initial release. Finally, the thermal properties and moisture content were measured to explore their participation in the burst release phenomena.

## 2 Materials and Methods

### 2.1 Materials

BSA Fraction V, Tween 20, phosphate-buffered saline (PBS), and Resomer RG 752H (PLGA) were obtained from Sigma-Aldrich (Dorset, UK). Dichloromethane 99.8% HPLC grade, acetonitrile 99.8% HPLC grade, and trifluoroacetic acid (TFA) 99+ % were purchased from Thermo Fisher Scientific Inc. (Loughborough, UK). Deionized water was produced by a Milli-Q Integral system (Merck Millipore Ltd., Hertfordshire, UK).

### 2.2 Methods

#### 2.2.1 Microsphere Preparation by Spray Drying

The BSA-encapsulated PLGA microspheres were prepared using a two-fluid spray-drying method. Three formulations were prepared with different protein-to-polymer ratios (by weight): *A* (1:5), *B* (1:10), and *C* (1:15). The feed solution was an emulsion consisting of the BSA dissolved in water (25 mg/mL) as the aqueous phase and PLGA dissolved in dichloromethane (14–42 mg/mL) as the organic phase. The two phases were emulsified with an Ultra-Turrax T-18 homogenizer (IKA-Werke GmbH & Co. KG, Staufen, Germany) for 2 min at 15,000 rpm. The emulsion was subjected to spray drying at an inlet temperature of 50–55 °C using a mini spray dryer Buchi B-290 and an inert loop Buchi B-295 (BÜCHI Labortechnik AG, Flawil, Switzerland) in closed mode with a nitrogen flow rate of 6.5–8.5 L/min, with a feed rate of 1–2 mL/min, and a drying gas flow rate of 33–37.6 m^3^/h. The spray-dried microspheres produced were collected in parafilm-sealed glass vials and stored at 2–8 °C until further characterization. The spray-drying method was adapted from Baras et al., and all process parameters were optimized to maximize yield while preserving microsphere structure and BSA stability.^31^

#### 2.2.2 Protein Loading

5 mg of BSA-loaded spray-dried microspheres was dissolved in a mixture of ethyl acetate and Milli-Q water and agitated using a combination of plate shaker and occasional vortexing for 1–2 h. The infranatant was collected and analyzed by reversed-phase high-performance liquid chromatography (RP-HPLC) using a Shimadzu UFLC system (Shimadzu Corporation, Kyoto, Japan) composed of a Jupiter 5 μm C5 300 Å column 4.6 mm i.d. × 250 mm length (Phenomenex Inc., Torrance, CA, USA). A gradient elution of water with 0.1% TFA (*A*) and acetonitrile with 0.1% TFA (*B*) at a flow rate of 1 mL/min was performed as follows: *A*/*B* from 95:5 to 35:65 in 20 min with a 2 min recovery to initial conditions. An ultraviolet (UV) detector with an absorption wavelength of 280 nm and an injection volume of 100 μL were used. A linear calibration plot for BSA was obtained over the range 15.625–2000 μg/mL (*n* = 8, *R*^2^ = 1, LOD = 7.80 μg/mL, and LOQ = 26 μg/mL). The method was adjusted from Umrethia et al., and validation was carried out following The International Council for Harmonisation of Technical Requirements for Pharmaceuticals for Human Use (ICH Q2 (R1)).^32^ The actual BSA loading was calculated using the following equation
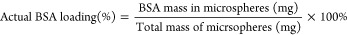
2

#### 2.2.3 In Vitro BSA Release

10 mg of BSA-loaded spray-dried microspheres was dispersed into 1 mL of dissolution media (PBS with 0.02% Tween 20) in polypropylene tubes with a hinged lid (Eppendorf AG, Hamburg, Germany) and incubated at 37 °C. At each time point (day 1, day 2, and then weekly for 8 weeks), the samples were centrifuged at 3000 RCF for 10 min; all the supernatant was collected and replaced with fresh media. The amount of BSA released was determined by RP-HPLC as described in Section 2.2.2.

#### 2.2.4 Particle Morphology by Scanning Electron Microscopy

The morphology of the BSA-loaded PLGA microspheres was examined before incubation, on day 2, day 7, day 30, and day 60, by a Philips XL30 ESEM FEG (Hillsboro, OR, USA) operating at 10 kV under high vacuum. A small amount of each sample was spread over double-sided tape on a sample holder. Scanning electron microscopy (SEM) images were taken at 2500, 7500, and 12,000× magnification.

#### 2.2.5 Particle Size and Size Distribution by Laser Diffraction

Particle size and size distribution were measured via laser diffraction using a Sympatec HELOS detector equipped with a RODOS dry disperser and a VIBRI feeder (Sympatec GmbH, Clausthal-Zellerfeld, Germany). 50 mg of sample was placed on the VIBRI feeder and fed through the RODOS disperser at a pressure of 4 bar and a measuring range between 0 and 175 μm. Measurements were taken in triplicates using PAQXOS 5.0 software and presented as volume mean diameter (VMD), *D*_10_, *D*_50_, *D*_90_, and span ± standard deviation. The span of the microsphere size distribution can reflect the dispersity of particles and was calculated using the following equation
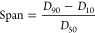
3

#### 2.2.6 Thermal Properties by Differential Scanning Calorimetry

The thermal properties of BSA-loaded PLGA microspheres were examined by a differential scanning calorimetry (DSC) instrument TA Q200 (TA Instruments, New Castle, DE, USA). 2–3 mg of samples were loaded in a Tzero low-mass aluminum pan. Temperatures were ramped between −20 and 90 °C at a rate of 5 °C/min under a nitrogen airflow of 50 mL/min in triplicates. All samples were subjected to a heat/cool/heat cycle. The analysis and thermograms were generated using TA Universal Analysis 2000 software (version 4.5). The method was adjusted from Shi et al.^33^

#### 2.2.7 Residual Moisture Content by Thermogravimetric Analysis

5 mg of BSA-loaded PLGA microspheres was loaded onto a platinum pan and analyzed using thermogravimetric analysis (TGA) instrument Pyris 1 (PerkinElmer, Waltham, MA, USA). To obtain the full profile, samples were heated from 20 °C until the end of decomposition at a heating rate of 10 °C/min under a nitrogen flow of 20 mL/min. The method was adjusted from Wan et al., and the residual moisture content was determined by calculating delta Y between 50 and 120 °C.^29^

#### 2.2.8 Surface Area by Nitrogen Physisorption

For the determination of surface area, nitrogen physisorption isotherms were obtained using a NOVAtouch LX^2^ gas sorption analyzer (Quantachrome Instruments, Boynton Beach, FL, USA). Spray-dried particles (100 mg) were placed in a 9 mm cell and degassed for 24 h under vacuum at 5.1 kPa (38 Torr) to remove surface contamination and adsorbed species. To generate the isotherm, the cell containing the sample was placed on the surface analysis station with a dewar filled with liquid nitrogen underneath. The surface area was calculated based on the adsorption of nitrogen at 77.3 K (liquid nitrogen temperature) onto the surface of the microparticles at relative pressures (*P*/*P*_0_) ranging from 0.05 to 0.3 using the Brunauer–Emmett–Teller (BET) method.^34^ The specific surface area was calculated by dividing the surface area by the sample weight. Analysis was performed in triplicates, and the data are presented as mean ± SD.

#### 2.2.9 Quantitative Chemical Analysis by X-ray Photoelectron Spectroscopy and Hard X-ray Photoelectron Spectroscopy

X-ray photoelectron spectroscopy (XPS) and hard XPS (HAXPES) were carried out under ultrahigh vacuum conditions using a Kratos Axis Supra^+^ spectrometer (Kratos Analytical, Manchester, UK). For XPS, a monochromated Al K α X-ray source (1486.6 eV photoenergy, 15 kV, and 5 mA emission current) was used with the analysis area of approximately 700 μm × 300 μm; for HAXPES, a monochromated Ag Lα X-ray source (2984.3 eV photo energy, 15 kV, and 25 mA emission current) was used, with the same analysis area as XPS. It should be noted that XPS using Al K α X-rays typically provides information from the top 10 nm of any surface, while the information depth of HAXPES using Ag Lα X-rays is typically up to 20 nm. The XPS survey spectra were acquired at a pass energy of 80 eV and a binding energy (BE) from 1300 to −10 eV (0.5 eV step size and 300 ms dwell for two sweeps). The HAXPES survey spectra were acquired at a pass energy of 160 eV and a BE from 2800 to −10 eV (0.5 eV step size and 300 ms dwell for three sweeps). Both XPS and HAXPES high-resolution spectra were acquired using a 0.1 eV step size and 300 ms dwell. BE range and number of sweeps are peak dependent. Spectra were processed using Casa XPS version 2.3.25; the energy-dependent instrumental transmission was corrected for using the NPL transmission function correction,^35,36^ and the BE scale was referenced to the C 1s line of aliphatic carbon, fixed at 285.0 eV.

The XPS samples were prepared by adding spray-dried microparticles into three aluminum wells, followed by gentle tapping to allow particle settling. Pure PLGA and BSA were first dissolved in chloroform and ultrapure water, respectively, before being drop-cast onto clean silicon wafers. Prior to sample deposition, all substrates were thoroughly cleaned using a cycle of sequential sonication in isopropanol (20 min), ultrapure water (20 min), and again isopropanol (5 min), followed by a drying process using a stream of compressed air.

#### 2.2.10 Surface Analysis and Chemical Depth Profiling by Time-of-Flight Secondary Ion Mass Spectrometry

Time-of-flight secondary ion mass spectrometry (ToF-SIMS) 2D and 3D mapping was carried out using a TOF.SIMS 5 instrument (IONTOF GmbH, Münster, Germany). Secondary ion mass spectra were acquired in the positive ion polarity mode using a 30 keV Bi_3_^+^ primary ion beam delivering 0.19 pA. For depth profiling, a 10 keV Ar_2000_^+^ beam delivering 4 nA was used as the sputter gun with a sputtering area of 250 μm × 250 μm for the sample BSA/PLGA 1:5 and 200 μm × 200 μm for the other two samples (BSA/PLGA 1:10 and 1:15). A low-energy (20 eV) electron flood gun was employed to neutralize charge build up. The analysis area was 100 μm × 100 μm for all the ToF-SIMS surface analysis measurements. The samples were prepared by depositing spray-dried microparticles as well as pure PLGA and BSA raw materials onto copper tape (product AGG3397, Agar Scientific, Essex, UK) supported by silicon wafers.

For chemical depth profiling, sputtering fluence, *F*, was calculated using the following equation^37^
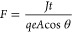
4where *J* is the average 10 keV Ar_2000_^+^ beam current measured before and after the measurements. *t*, *q*, and *e* are the sputtering time, the charge number on the argon cluster ion, and the elementary charge on an electron, respectively. *A* and θ are the raster areas on the surface and the incident angle of the argon cluster ion beam with respect to the surface normal, respectively. The fluence is calculated using θ = 0° regardless of the incident angle and is more appropriate for flat surfaces and large particles. The relative uncertainty in sputtering fluence is largely due to uncertainty in the sputtering area measured on the sample and the instability of the sputtering current. It is worth noting that any matrix effect in SIMS analysis may also affect the measured chemical depth profile.^38^

The sputtered depth normal to the incident ion beam (Δ*h*, nm) was estimated using the following equation
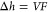
5where *V* is the sputtering yield volume (nm^3^/ion). Here, we assume that there is a constant sputtering yield volume, and the sample materials’ sputtering behavior is similar to polystyrene. Based on the literature values of the flat^39,40^ and spherical^41^ polystyrene surfaces, we estimate a sputtering yield volume *V* of 30 nm^3^/ion for the polymeric microparticle samples for the depth calculation. Seah et al. described that the useful depth profiling of particles, if there is no concern of material melting or degradation, may occur in the early stage of the sputtering process up to a depth equivalent to particle radius.^42^

To minimize contribution from the copper substrate and artifacts from sample topography, regions-of-interest (ROIs) were established to consider pixels within microparticles only. To produce the scatter plots in Figure 6, the images from the ROIs were down-binned by a factor of 4, and the intensity for each relevant secondary ion was averaged.

**Figure 1:**
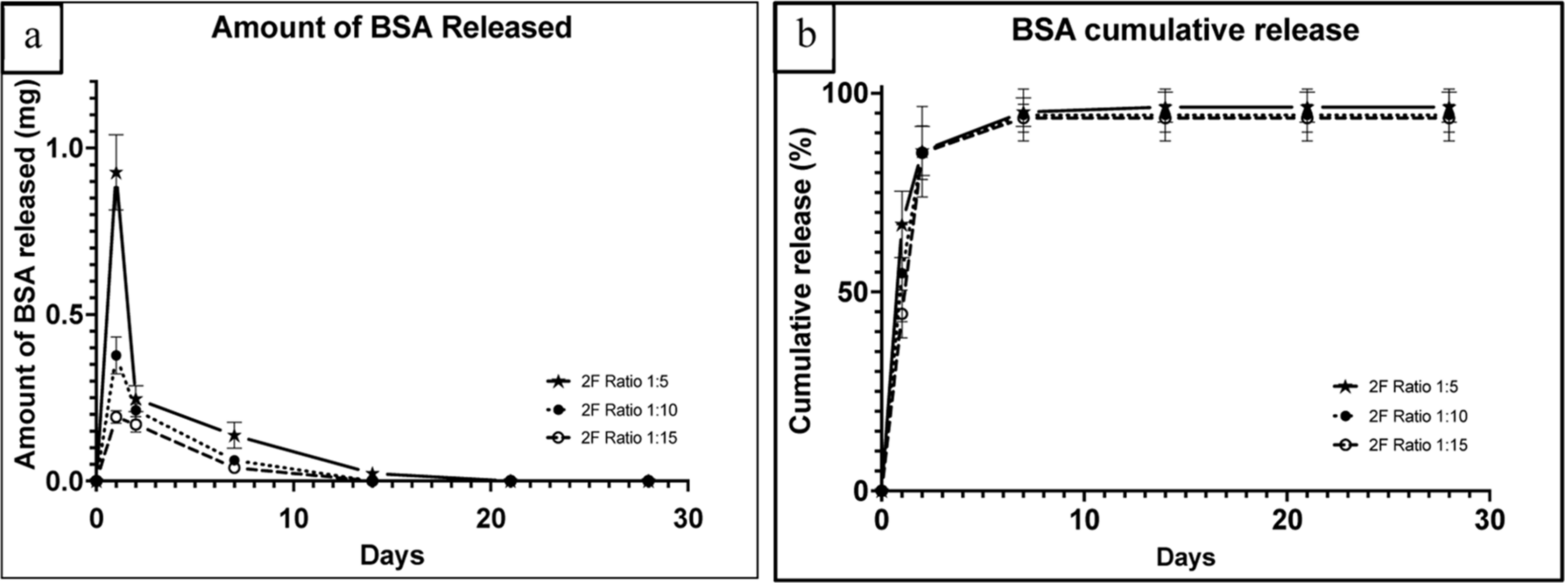
(a) BSA release from PLGA microspheres (10 mg) formulated with ratio 1:5, 1:10, and 1:15 (b) BSA cumulative release normalized to the actual BSA loading at each ratio (ratio 1:5 → 13.80%, ratio 1:10 → 6.86%, and ratio 1:15 → 4.30%). Error bars represent the standard deviation (*n* = 3).

**Figure 2:**
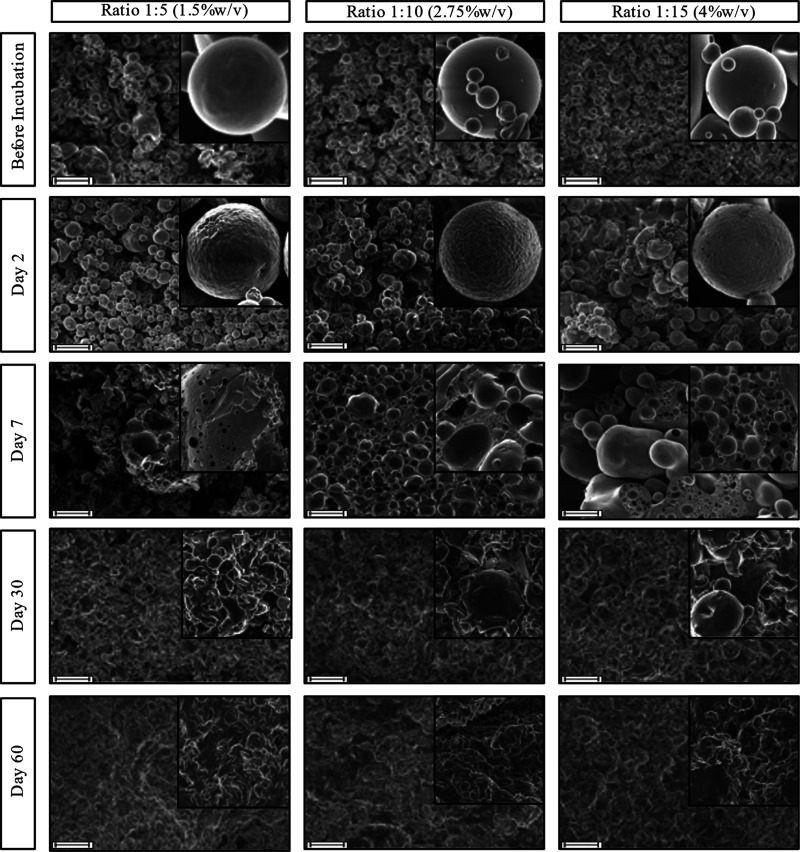
SEM images of the three formulations at 2.50 K× magnification at different time points: before incubation and after incubation at 37 °C in PBS for 1, 2, 7, 30, and 60 days (scale bar = 10 μm). Images in the insets are from the same formulations at 12.00 K× magnification.

**Figure 3:**
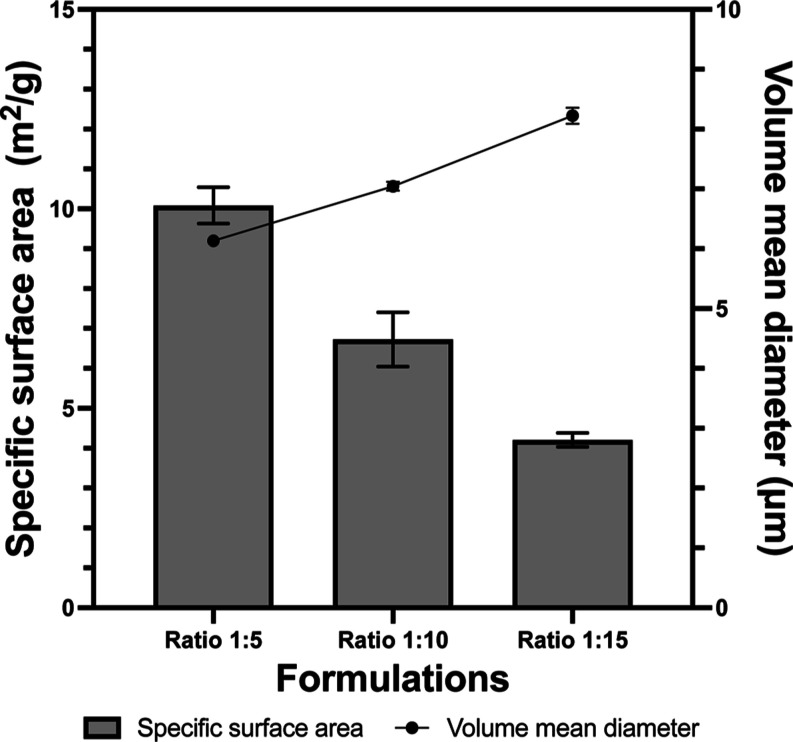
Specific surface area measured by gas physisorption analysis and VMD measured by laser diffraction of three formulations (BSA/PLGA ratio 1:5, 1:10, and 1:15). Error bars represent standard deviation of the mean (*n* = 3).

**Figure 4:**
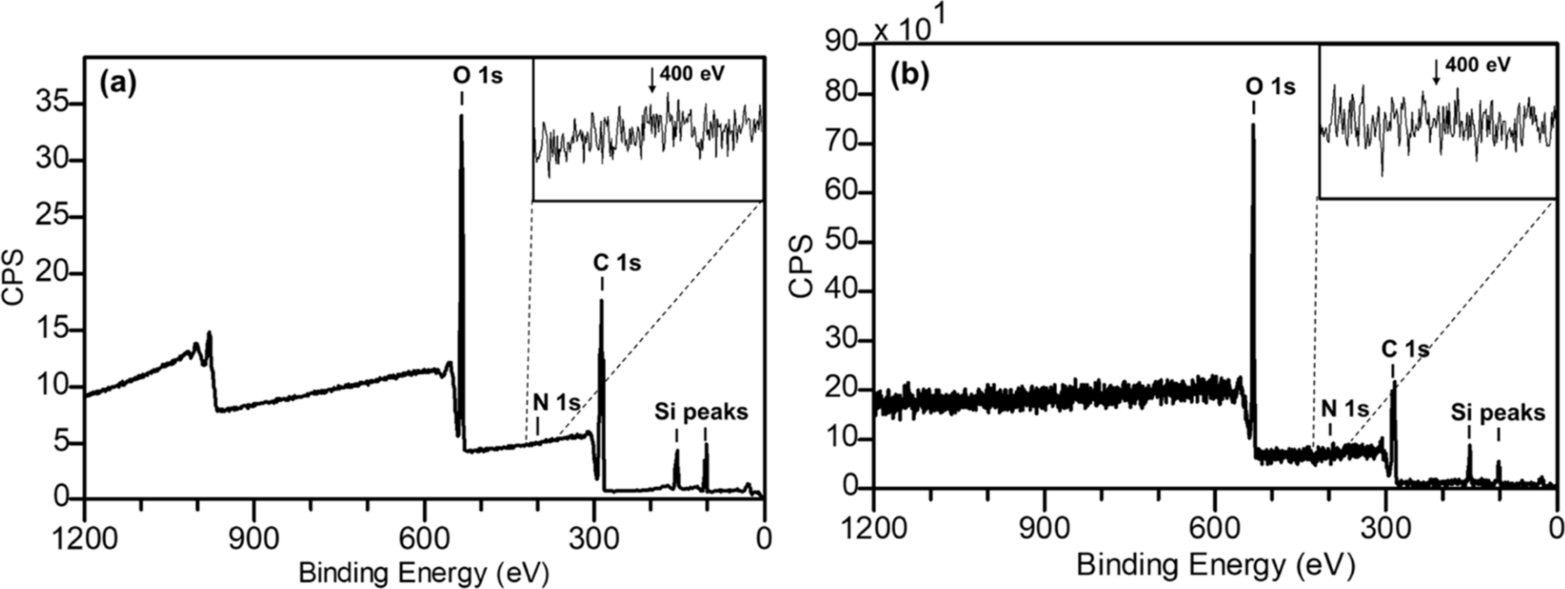
Representative (a) XPS and (b) HAXPES survey spectra of sample C and the polymeric microparticle formulation with a BSA/PLGA ratio of 1:15. Inset images are high-resolution N 1s spectra recorded by XPS and HAXPES.

**Figure 5:**
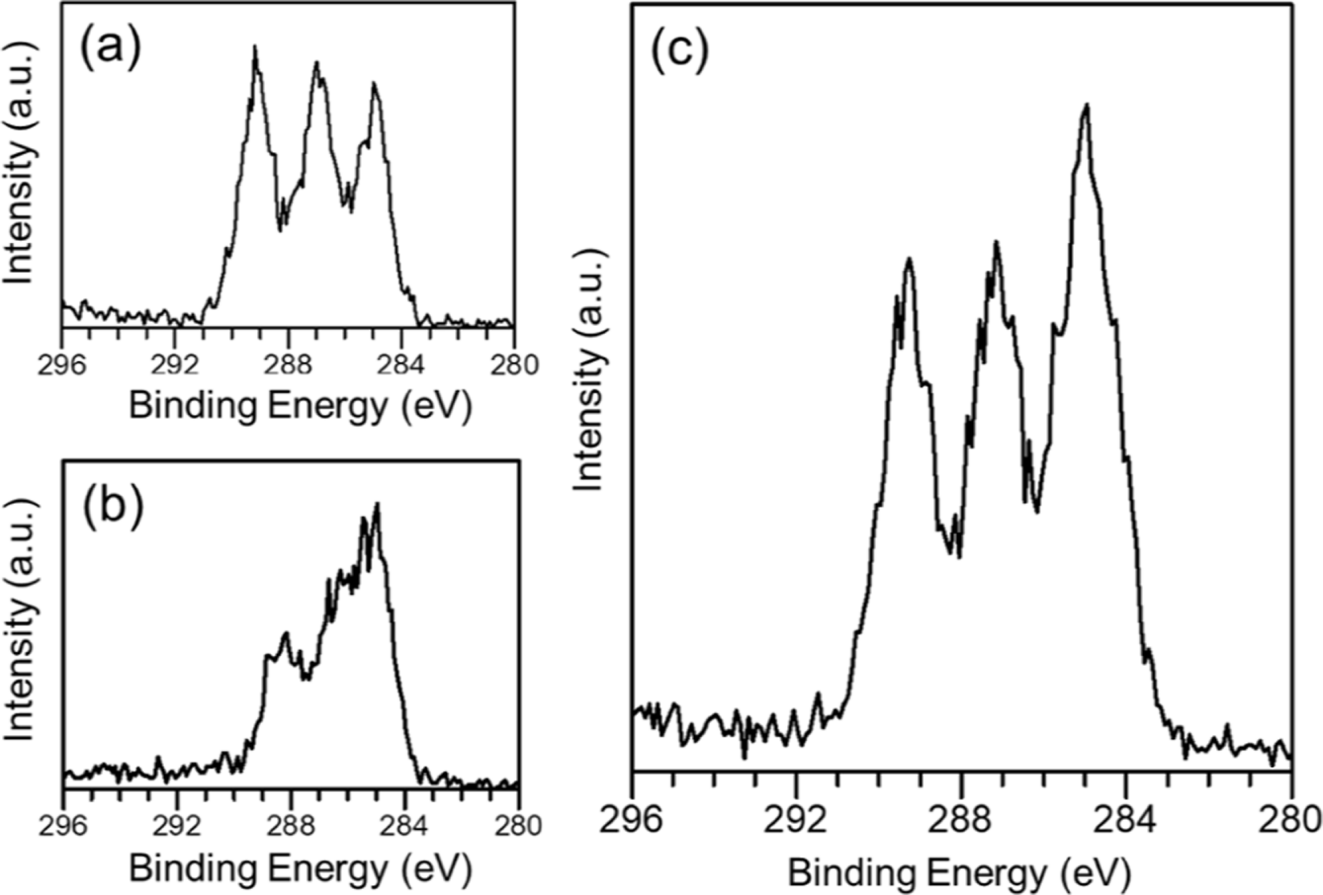
Representative HAXPES high-resolution C 1s of (a) pure PLGA, (b) pure BSA protein, and (c) sample C, and the polymeric microparticles with a BSA/PLGA ratio of 1:15.

**Figure 6:**
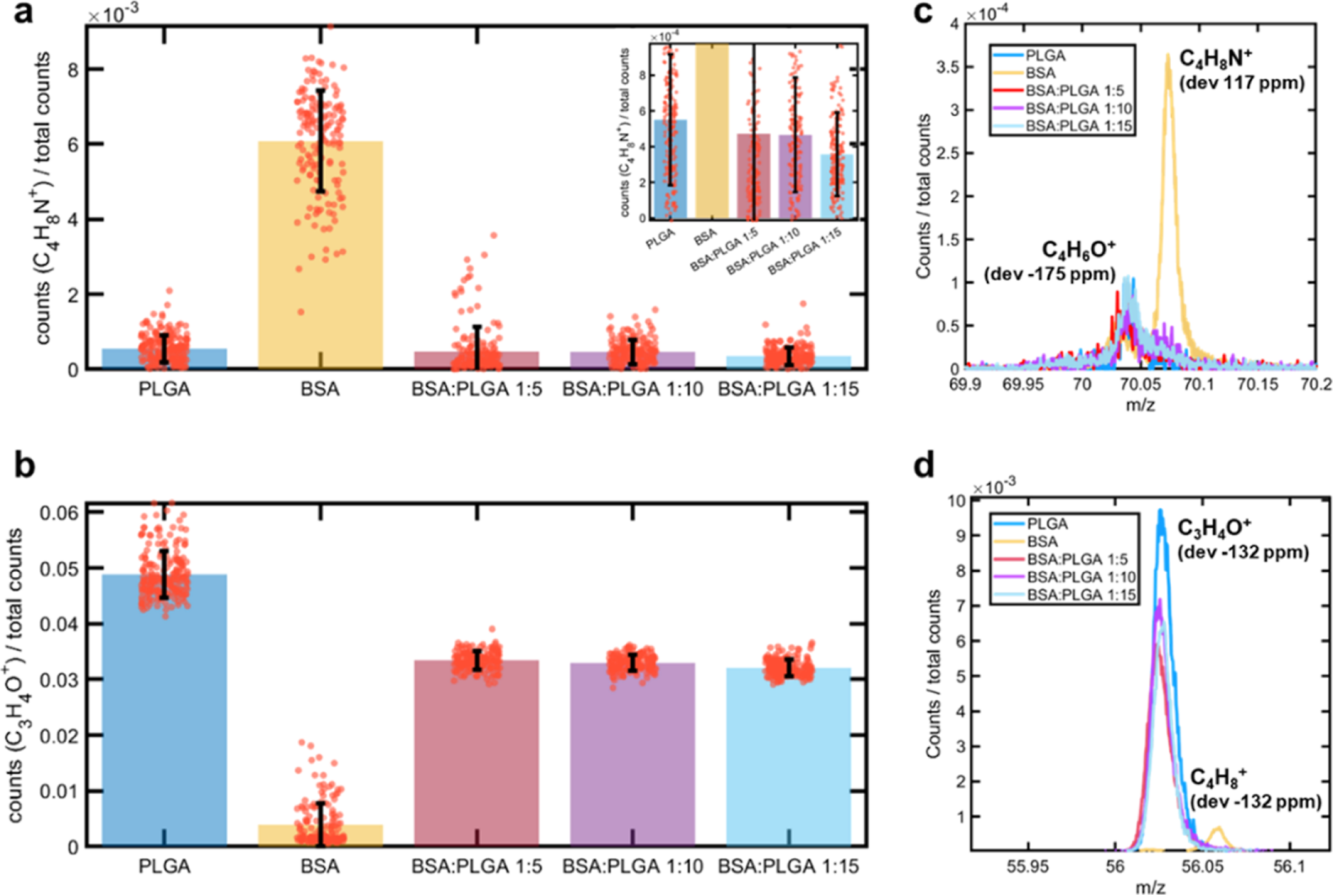
ToF-SIMS surface chemical analysis of the particles sample, pure PLGA and BSA: normalized ion intensities of the characteristic peaks of (a) BSA protein (C_4_H_8_N^+^) and (b) PLGA (C_3_H_4_O^+^), together with representative normalized ion peaks of (c) C_4_H_8_N^+^ and (d) C_3_H_4_O^+^. Each data point corresponds to a pixel in a surface mapping data set.

#### 2.2.11 Statistical Analysis

GraphPad Prism version 10 (GraphPad Software, San Diego, CA, USA) was used to carry out a one-way analysis of variance. An alpha value of 0.05 was used to determine the significance.

## 3 Results and Discussion

### 3.1 Release of BSA from PLGA Microspheres

All three PLGA formulations containing BSA exhibited a burst release profile with no detectable amount of protein beyond day 14, as shown in Figure 1. At day 1, formulations that contained a higher PLGA concentration showed a smaller burst effect, for example, (44.6 ± 3.7) % in the 1:15 formulation in comparison with (67.3 ± 8.2) % in the 1:5 formulation. However, most of the BSA (85%) was released after the second day from all three formulations, suggesting that the protein may have rapidly diffused out of the microspheres or it was present on or close to the surface. After the initial burst, the protein release declined steeply reaching amounts below 200 μg after day 7 (Figure 1a). More than 94% of loaded BSA was released by the end of the release studies in all formulations (Figure 1b).

The phenomenon of burst release from spray-dried BSA-encapsulated PLGA microspheres was observed in previous studies.^31,43,44^ Mok and Park reported a decrease of burst release from (74.5 ± 3) % to (18 ± 3) % when increasing polymer concentration by changing the BSA/PLGA ratio from 1:5 to 1:52.^44^ However, using higher polymer concentrations can have negative consequences such as dosing problems and high viscosity if high doses of API are required to provide a therapeutic effect. Giunchedi et al. (2001) observed burst release within the first 2 days ranging from 40% to 80% depending on the emulsifier used during w/o emulsification.^43^ Baras et al. (2000) also observed a burst release effect of (92.6 ± 3.4) % in their initial spray-dried microsphere formulations.^31^ Different groups employed various approaches to reduce burst release with a focus on modulating the spray-drying process parameters such as feed rate, inlet temperature, and atomization rate. In the current study, the focus is on formulation parameters, more specifically, the BSA/PLGA ratio, which is one of the most critical factors affecting the injectable formulation potential for translation (dose size and viscosity). Multiple characterization techniques were utilized to explore the impact of the physical and chemical properties of the microspheres on the burst release observed.

### 3.2 Morphology Analysis, Microsphere Size Distribution, and Surface Area

First, we investigated the morphology evolution of the microsphere samples, as shown in Figure 2. Microsphere surface morphology as well as pore formation can provide useful insights into PLGA-controlled release mechanisms.^45^ Before incubation, the microspheres of the three formulations had a nonporous smooth surface and an average size below 10 μm. By day 2, when almost 85% of the protein had been released (Figure 1), the microspheres appear to retain their original structure, but they exhibited a wrinkled surface and emergence of pores in all formulations, indicating diffusion of protein out through those channels. More pores appear on the 1:15 microspheres potentially enabling BSA to diffuse out, whereas the 1:5 formulation have visible “craters”, suggesting hydrolysis of the ester bonds and potential protein presence closer to the surface rather than the core.^46^ A possible explanation for the wrinkling is that the outer layer of PLGA microspheres absorbs water and swells, whereas the inner layers stay intact.^47^ To compensate for the increase in the surface area, wrinkles are formed. By day 7, most of the microspheres in formulation 1:5 lost their spherical shapes due to erosion, and large pores appeared on their surface. In the formulations 1:10 and 1:15, the microspheres aggregated. The precipitated PLGA seems to form a macromolecular network structure accommodating PLGA microspheres in a polymeric skeleton.^48^ After 30 days of incubation, it was evident that as the ratio of PLGA increased (from 1:5 to 1:15) in the formulations, more microspheres retained their spherical structure with potential for further release. By day 60, all three microsphere formulations were fully eroded.

Particle size and the size distribution of the samples were measured by laser diffraction. As the polymer concentration in the spray dryer feed increased, the VMD also increased, while the span remained similar. Microspheres smaller than 10 μm can be prone to diffusion-controlled API release, resulting in the burst profile observed, i.e., all the protein diffuses out before reaching erosion stages.^20^ There was no significant difference between the spray-dried PLGA control (8.2 ± 0.4) μm and the formulations (*p* = 0.92). Formulation 1:5, 1:10, and 1:15 had a VMD of (6.13 ± 0.06) μm, (7.04 ± 0.07) μm, and (8.2 ± 0.1) μm, respectively. The small size difference (statistically significant, *p* = 5 × 10^–7^) observed in the formulations may be responsible for the initial reduction of burst release on day 1 but did not seem to have an effect on the long-term release profile. Larger microspheres can exhibit sigmoidal release profile governed by both erosion and diffusion, causing a delay in release.^26,49^ However, for long-acting injectable formulations, smaller particle size could be favorable to avoid injection site pain.^50^

The specific surface area decreased as a function of the PLGA concentration in the feed (Figure 3). The decrease in the specific surface area was directly proportional to the increase in the laser-diffraction-measured particle size. Formulation 1:5, 1:10, and 1:15 had mean specific surface areas of 9.91, 6.73, and 4.21 m^2^/g, respectively (statistically significant, *p* = 0.004). Similar specific surface area for PLGA microspheres analyzed using the BET method was reported by other groups.^51,52^ Compared to the literature values (Gupta and Ahsan, 2011, Semete et al., 2010), a higher burst release observed in our formulations can be linked to the measured surface areas. For example, with 1:5, the surface area (9.91 m^2^/g) was the highest among the three formulations, and it had the largest burst release on day 1. This could be due to the higher chance of interaction between the release media and BSA present near the surface.^53^

### 3.3 Quantitative Chemical Analysis, Surface Analysis, and Chemical Depth Profiling

To investigate whether the burst release was caused by BSA being present on the surface of the microspheres (not properly encapsulated) or by the encapsulated protein rapidly diffusing out, XPS and HAXPES were employed to perform quantitative chemical analysis on spray-dried protein-loaded PLGA microparticles. XPS analysis provides chemical information on samples’ uppermost surface, whereas HAXPES extends the sampling depth below the topmost surface to reveal buried materials by increasing photoelectron kinetic energies and, thus, escape depths.^54^ Complementary to gas-cluster-ion-sputtering-assisted chemical depth profiling, HAXPES can reveal quantitative chemical information without removing any surface material. Table 1 shows the homogeneous-equivalent atomic concentrations of the three microparticle samples (BSA/PLGA ratio of 1:5; 1:10, and 1:15) as well as pure PLGA and BSA. Calculations of atomic concentration were performed using XPS and HAXPES survey spectra (Figure 4 and in Figures S1 and S2) and average matrix relative sensitivity factors from Seah et al. for XPS^55^ and Cant et al. for HAXPES.^56^ Both XPS and HAXPES analyses revealed that there were no detectable protein signals on the topmost surface or under buried layers (up to approximately ∼20 nm) of any of the three particle samples. XPS analysis shows the presence of carbon, oxygen, and silicon signals in these particle samples with no significant differences in their homogeneous-equivalent elemental compositions. HAXPES analysis shows results similar to those measured by XPS, suggesting negligible changes in the chemical composition with increasing sampling depth. All particle samples also exhibited ∼10 at. % of siloxane contaminations at BE values of ∼103.5 eV. It is known that siloxane-based products, such as poly(dimethylsiloxane), are among most common contaminants on many surfaces that can be observed in XPS and SIMS analysis.^57^Figure 5 shows high-resolution C 1s spectra of particle sample C (BSA/PLGA = 1:15), pure PLGA and BSA measured by HAXPES. The C 1s spectrum of the particle sample exhibited peaks consistent with PLGA,^56,58^ i.e., C–C/C–H at ∼285.0 eV, C–O at ∼287.0 eV, and C (=O)–O at ∼289.0 eV, and again, the relatively higher hydrocarbon signals are likely associated with certain carbon-based surface contamination. These XPS and HAXPES measurements suggest a surface enrichment of PLGA with no detectable BSA protein component in all three spray-dried-loaded microparticles (up to ∼20 nm in depth).

**Table 1:** XPS and HAXPES Homogeneous-Equivalent Elemental Compositions of Pure PLGA, Pure BSA, and Spray-Dried Polymer Microparticles with Variable Formulations in at %

code	description	C at. %		O at. %		N at. %		S at. %		Si at %	
		XPS	HAXPES	XPS	HAXPES	XPS	HAXPES	XPS	HAXPES	XPS	HAXPES
PLGA	pure PLGA	63.1	63.0	36.9	37.0	n.a.	n.a.	n.a.	n.a.	n.a.	n.a.
BSA^a^	pure BSA protein	63.9	59.9	17.4	18.8	15.5	17.3	1.7	1.9	n.a.	n.a.
A	microparticles (BSA/PLGA = 1:5)	57.3	57.0	30.8	32.4	n.a.	n.a.	n.a.	n.a.	11.9	10.6
B	microparticles (BSA/PLGA = 1:10)	57.6	56.6	32.0	32.3	n.a.	n.a.	n.a.	n.a.	10.4	11.1
C	microparticles (BSA/PLGA = 1:15)	57.4	54.1	31.9	36.6	n.a.	n.a.	n.a.	n.a.	10.7	9.3

a The BSA sample also contains small quantity of salt (i.e., NaCl) detected by XPS and HAXPES.

Complementary to XPS/HAXPES analysis, gas cluster sputtering-assisted ToF-SIMS was employed to investigate the internal distribution of the key components in various organic particles, including protein-loaded PLGA drug carriers^30^ and polymeric core–shell nanoparticles.^41^ ToF-SIMS provides high-resolution chemical analysis of any surfaces, whereas argon cluster sputtering has been widely used to perform controlled, sequential removal of surface material allowing chemical analysis of buried layers.^59^Figure 6 shows normalized intensities and respective ToF-SIMS peaks for secondary ions related to BSA protein (C_4_H_8_N^+^) and PLGA (C_3_H_4_O^+^) in all three samples (BSA/PLGA ratio = 1:5; 1:10; and 1:15) in comparison to the pure PLGA and BSA. Specifically, the ion signals of C_4_H_8_N^+^ (*m*/*z* 70) and C_3_H_4_O^+^ (*m*/*z* 56) were selected as the ion from the proline fragment in BSA^60^ and the [M–O]^•+^ ion from the lactic acid monomer fragments in PLGA,^58^ respectively. The positive ion spectra of the particle surfaces contain only the characteristic signals for PLGA (Figure 6), and only background (due to noise or metastable) from the C_4_H_6_O^+^ peak is observed (Figure 6a,c). Hence, we assume that PLGA and BSA are phase-separated in the microsphere samples. The ToF-SIMS data also confirmed the presence of siloxanes, with a series of characteristic peaks (including the end group SiCH_3_^+^) being detected. The depth profiling data shows that these are confined to the top surface and are very likely due to surface contamination, which is common for these types of samples.^61^ Considering the difference in measurement sensitivity, i.e., detection limit in SIMS (ppm) versus that in XPS/HAXPES (∼0.1 at. %),^62^ these results are in agreement with the XPS/HAXPES analysis.

**Figure 7:**
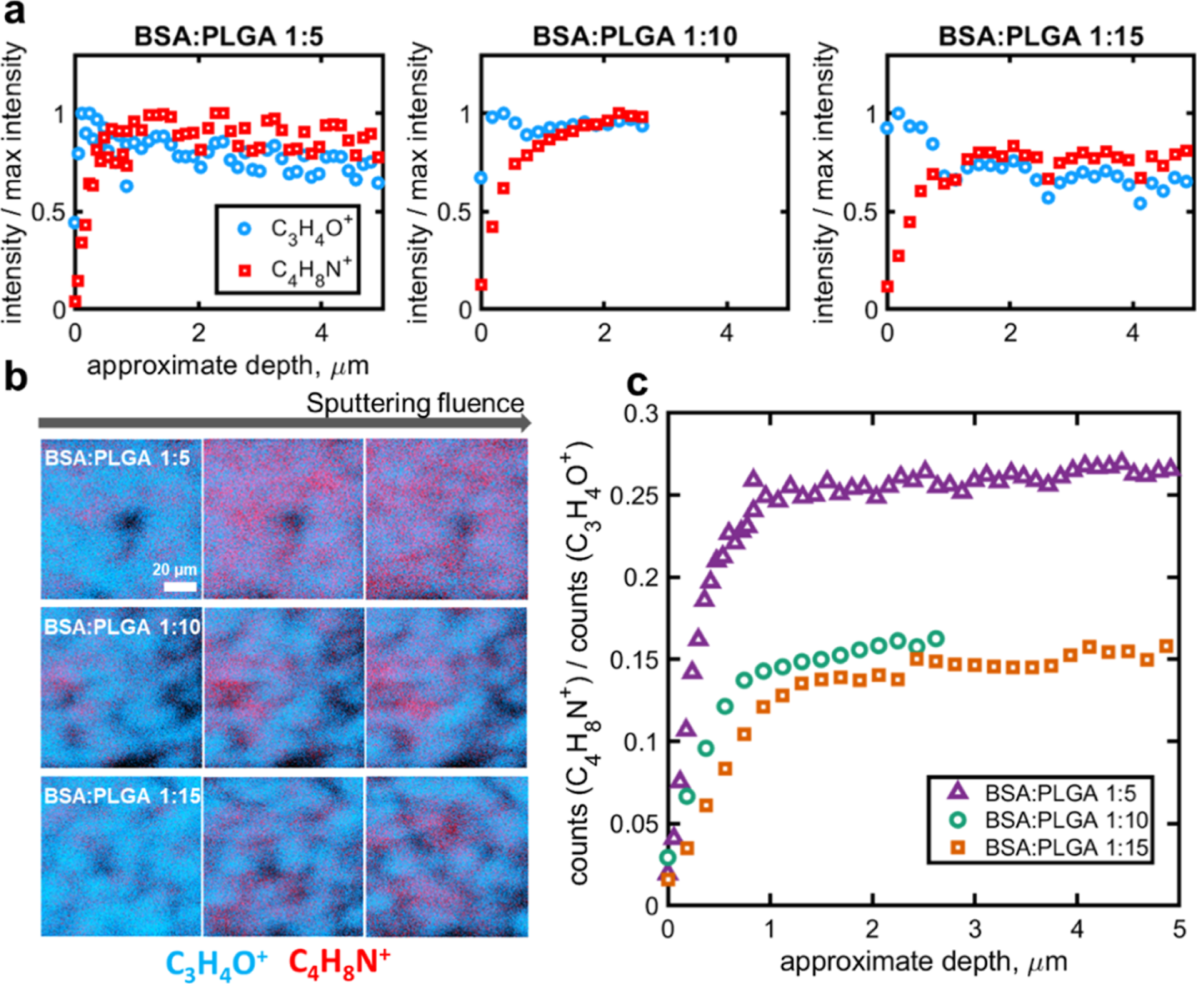
Chemical depth profiling of particle formulations using argon cluster sputtering-assisted ToF-SIMS analysis: (a) BSA/PLGA = 1:5, BSA/PLGA = 1:10, and BSA/PLGA = 1:15; (b) ToF-SIMS image overlay showing BSA protein (C_4_H_8_N^+^) and PLGA (C_3_H_4_O^+^) with increasing sputtering fluence; and (c) comparison of the ion signal ratio between C_4_H_8_N^+^ and C_3_H_4_O^+^ for all three formulations.

Figure 7a shows the normalized ion intensities of both BSA (C_4_H_8_N^+^) and PLGA (C_3_H_4_O^+^) signals as a function of the estimated sputtering depth for all particle samples. Strikingly, despite variation in their formulations, these microparticle samples show a similar trend: an enrichment of PLGA on the surface, followed by a plateau of intensity for the remainder of the depth; in contrast, there is a drastic increase of the BSA signal upon sputtering until it reaches a plateau at approximately 1 μm in depth from the surface. Figure 7b illustrates this with chemical maps at different sputtering fluences. Figure 7c shows a depth profiling of the ion signal ratio between BSA (C_4_H_8_N^+^) and PLGA (C_3_H_4_O^+^) at various sputtering depth. All three ratio profiles have a similar trend of a sharp increase, followed by a plateau, with differences depending on the protein loading in samples. This is indicated by (i) an increase in the intensity ratio of (C_4_H_8_N^+^)/(C_3_H_4_O^+^) with increasing BSA content, at early stage of the etching process (up to ∼1 μm) and (ii) increasing BSA/PLGA signal ratio with increasing BSA loading in the plateau region (after 1 μm). Based on laser diffraction measurements (Figure 3), the average radius of these three particle samples ranged between ∼3.1 and ∼4.1 μm. In all samples, the most changes in chemical profiles were observed up to ∼1 μm in depth, i.e., closer to the surface of the microparticles rather than their core. The comprehensive chemical analysis of the BSA/PLGA system shows the capacity for understanding the distribution of key components within the complex architecture of microsized therapeutics which can provide potential links to their drug release profiles. For example, since there is no evidence of BSA surface enrichment, the burst release observed in these particle samples is likely from the subsurface. Also, the higher BSA/PLGA signal (for the BSA/PLGA 1:5 formulation) can be linked to the larger burst release observed on day 1 of the release study (Figure 1).

### 3.4 Thermal Properties and Moisture Content

**Figure 8:**
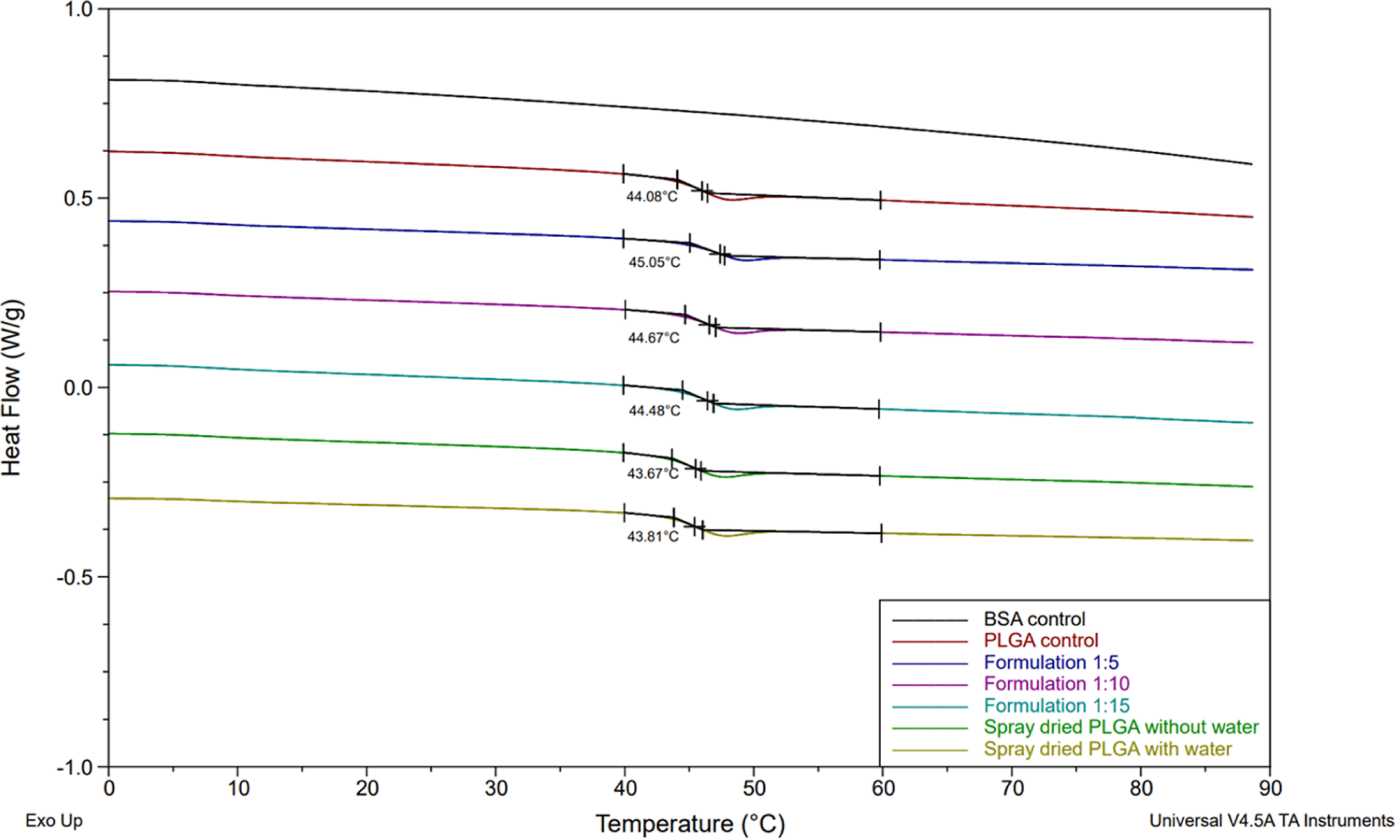
DSC thermograms for BSA, unprocessed PLGA, spray-dried PLGA with/without water and formulation 1:5, 1:10, and 1:15. The temperatures underneath the curves indicate the onset *T*_g_ values.

From the chemical depth profiling, it is evident that BSA is distributed within the PLGA matrix coated with a PLGA layer of at least 20 nm. The distribution of a large and structurally complex protein such as BSA within a polymer matrix can potentially affect the glass-transition temperature. Therefore, DSC was used to assess the thermal behavior of PLGA microspheres. All samples exhibited endothermic events corresponding to the glass-transition temperature of PLGA. The average glass-transition temperature was (44.6 ± 0.7), (44.4 ± 0.4), and (44.2 ± 0.5) °C for formulations 1:5, 1:10, and 1:15, respectively (Figure 8). The smaller the difference between the *T*_g_ and the temperature the formulation is exposed to (e.g., physiological environment 37 °C), the bigger the burst release.^63^

To determine whether BSA has any effect, we tested spray-dried PLGA without BSA in the aqueous phase was tested. There was no significant difference between the BSA-loaded and nonloaded formulations, suggesting that BSA has a neutral plasticizing effect.^64^ To assess the effect of residual water, spray-dried PLGA without water in the composition (only DCM) of the feed was analyzed. Similar results to the formulations 1:5, 1:10, and 1:15 were observed, deeming it necessary to explore the moisture content of the formulations.

Formulations 1:5, 1:10, and 1:15 had a very low relative moisture content of (0.21 ± 0.01) % (w/w), (0.07 ± 0.01) % (w/w), and (0.05 ± 0.01) % (w/w), respectively. Other groups also observed a very low moisture content in spray-dried PLGA microparticles. Sivadas et al. had (0.49 ± 0.01) %, w/w in their BSA-loaded PLGA microparticles, and Wan et al. observed relative moisture content values between 0.45% and 0.55% in their loaded PLGA microparticles.^28,65^ The moisture content can be significantly affected by excipients and the PLGA: excipient ratio.^66^ Moisture content has been identified as a considerable parameter in altering glass-transition temperature and having an effect on the drug release profile.^67^ According to a previous work, when the moisture content increased from (0.82 ± 0.04) % to (2.61 ± 0.10) % (w/w), the glass-transition temperature decreased from (42.8 ± 0.6) to (28.6 ± 0.5) °C.^68^ However, the results of the current study, where such changes in *T*_g_ were not observed, suggest that the residual moisture in the PLGA microparticles is probably not the driving factor that led to the burst release phenomenon.

## 4 Conclusions

The generated matrix-type microspheres with a surface layer of PLGA and buried BSA exhibited a burst release profile. Increasing the PLGA concentration did not mitigate the burst effect. The BSA loading beneath the surface seems to facilitate pore formation in the particles, leading to enhanced diffusion through the initial capping layer of PLGA. Our work is the first to uncover, using advanced analytical techniques, that the drug just beneath the surface layer could be the primary driver for burst release. By combining cutting-edge methods with traditional bulk powder techniques, we provide chemical profiling evidence linked directly to the release profile, a novel approach not previously explored, particularly in PLGA microspheres developed via spray-drying. Optimization of process and formulation parameters can improve encapsulation and achieve the desired release profiles. Spray-drying parameters and the constituents of the feed have a direct effect on the burst release phenomenon and on the overall release profile. For example, reducing the rate of solvent evaporation or increasing the viscosity of the constituents could help prevent high concentrations of BSA from migrating from deeper locations within the microsphere to just beneath the PLGA surface, potentially reducing the burst effect. Also, the BSA/PLGA ratio affects protein location within the polymer matrix, leading to different degrees of burst release. Therefore, XPS, HAXPES, and argon cluster sputtering-assisted ToF-SIMS hold potential to be highly complementary techniques for the characterization of long-acting injectables to gain deeper understanding on their structure–performance relationships and the impact of the manufacturing process on product performance and quality. These techniques are currently undergoing integration and further validation to facilitate their prospective routine application in the analysis or characterization of complex PLGA-based therapeutics.

## Supporting Information Available

Representative XPS and HAXPES survey spectra of the polymeric microparticle formulations with BSA/PLGA ratios of 1:5 and 1:10 (DOC) (PDF)
