# Supplementary material for: New Insights on the Burst Release Kinetics of Spray-Dried PLGA Microspheres
Source: Mol Pharm. 2024 Oct 25;21(12):6245–56. doi: 10.1021/acs.molpharmaceut.4c00686 (PMC11615953; doi:10.1021/acs.molpharmaceut.4c00686)
Supplement: Supplementary file 1 — mp4c00686_si_002.pdf [file mp4c00686_si_002.pdf]

Supporting Information

New insights on the burst release kinetics of spray-dried PLGA microspheres

Kyprianos Michaelides[1], Mohamad Anas Al Tahan[1], Yundong Zhou[2], Gustavo F. Trindade[2], David J. H. Cant[2], Yiwen Pei[2], Pawan Dulal[3], Ali Al-Khattawi[1]*

[1]School of Pharmacy, Aston University, Birmingham, B4 7ET, United Kingdom

[2]Chemical and Biological Sciences Department, National Physical Laboratory, Hampton Road, Teddington, TW11 0LW, United Kingdom

[3]aVaxziPen Limited, Milton Park, Abingdon, Oxfordshire, OX14 4SA, United Kingdom

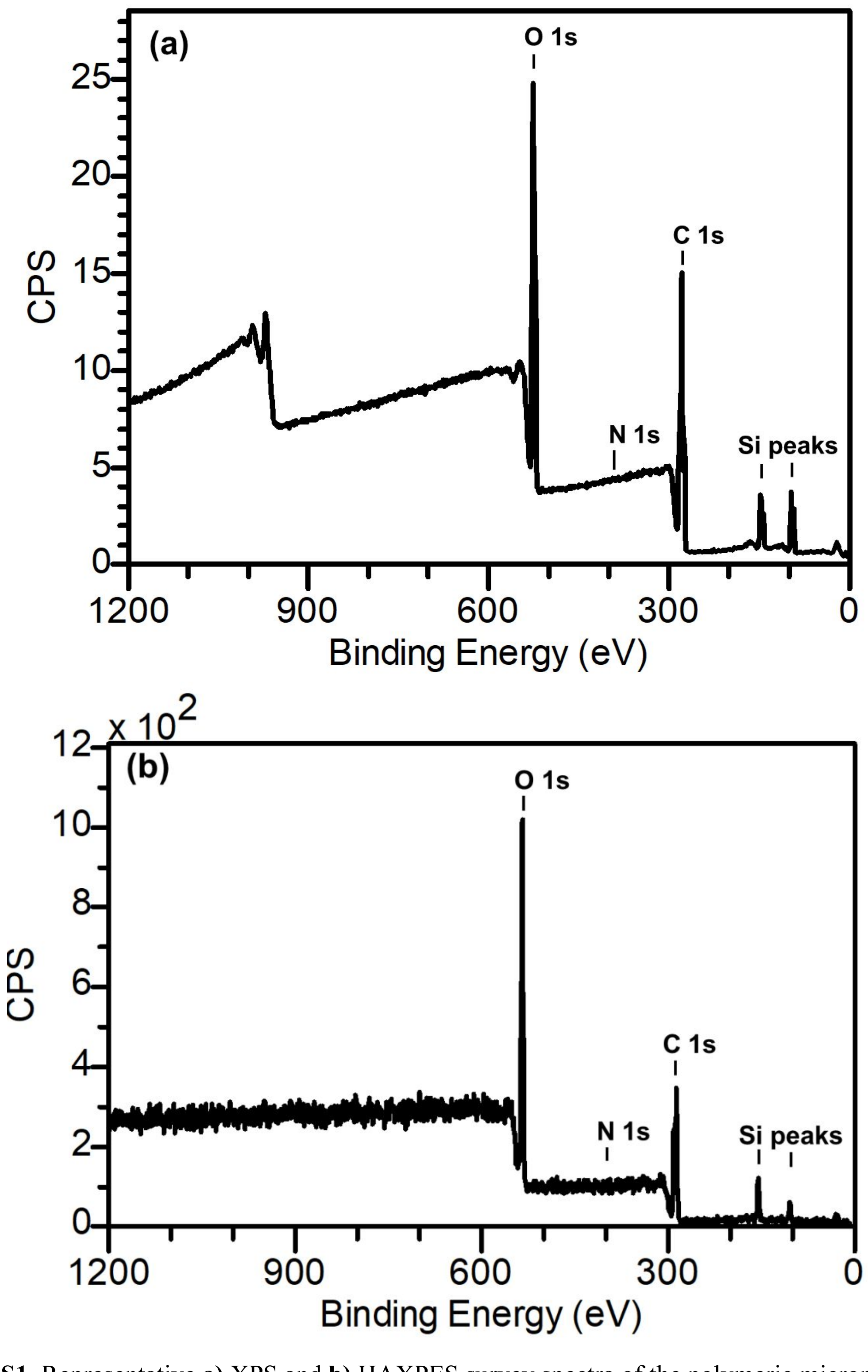

Figure S1. Representative **a)** XPS and **b)** HAXPES survey spectra of the polymeric microparticle formulation with a BSA:PLGA ratio of 1:5.

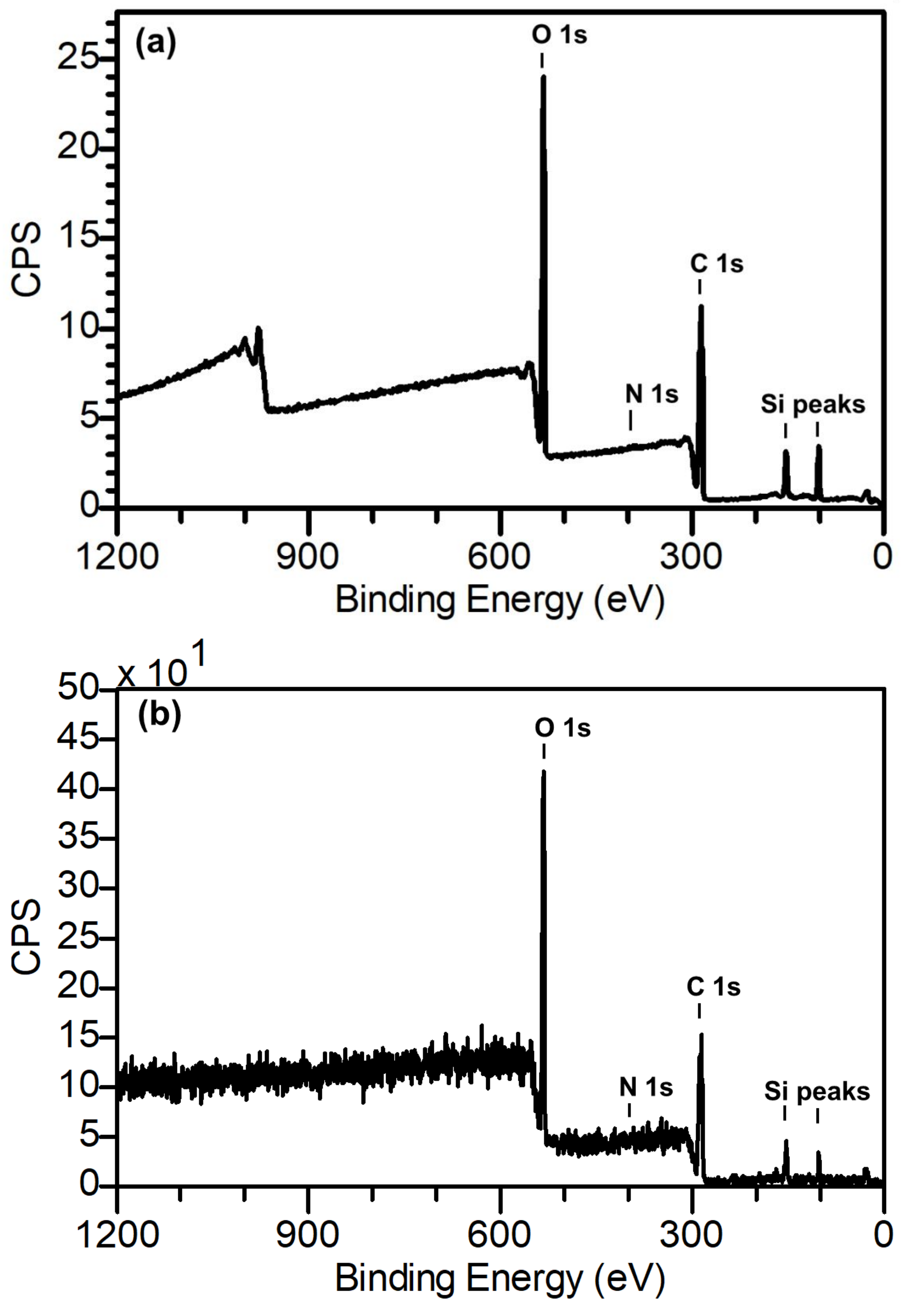

Figure S2. Representative **a)** XPS and **b)** HAXPES survey spectra of the polymeric microparticle formulation with a BSA:PLGA ratio of 1:10.
